# A multipurpose TNM stage ontology for cancer registries

**DOI:** 10.1186/s13326-022-00260-w

**Published:** 2022-02-22

**Authors:** Nicholas Charles Nicholson, Francesco Giusti, Manola Bettio, Raquel Negrao Carvalho, Nadya Dimitrova, Tadeusz Dyba, Manuela Flego, Luciana Neamtiu, Giorgia Randi, Carmen Martos

**Affiliations:** European Commission Joint Research Centre, Ispra, Italy

**Keywords:** Cancer registry, Ontology convergence, OWL, Unified TNM ontology, TNM stage tool, Data validation

## Abstract

**Background:**

Population-based cancer registries are a critical reference source for the surveillance and control of cancer. Cancer registries work extensively with the internationally recognised TNM classification system used to stage solid tumours, but the system is complex and compounded by the different TNM editions in concurrent use. TNM ontologies exist but the design requirements are different for the needs of the clinical and cancer-registry domains. Two TNM ontologies developed specifically for cancer registries were designed for different purposes and have limitations for serving wider application. A unified ontology is proposed to serve the various cancer registry TNM-related tasks and reduce the multiplication effects of different ontologies serving specific tasks. The ontology is comprehensive of the rules for TNM edition 7 as required by cancer registries and designed on a modular basis to allow extension to other TNM editions.

**Results:**

A unified ontology was developed building on the experience and design of the existing ontologies. It follows a modular approach allowing plug in of components dependent upon any particular TNM edition. A Java front-end was developed to interface with the ontology via the Web Ontology Language application programme interface and enables batch validation or classification of cancer registry records. The programme also allows the means of automated error correction in some instances. Initial tests verified the design concept by correctly inferring TNM stage and successfully handling the TNM-related validation checks on a number of cancer case records, with a performance similar to that of an existing ontology dedicated to the task.

**Conclusions:**

The unified ontology provides a multi-purpose tool for TNM-related tasks in a cancer registry and is scalable for different editions of TNM. It offers a convenient way of quickly checking validity of cancer case stage information and for batch processing of multi-record data via a dedicated front-end programme. The ontology is adaptable to many uses, either as a standalone TNM module or as a component in applications of wider focus. It provides a first step towards a single, unified TNM ontology for cancer registries.

## Background

### Population-based cancer registries

Population-based cancer registries (CRs) play a critical role in the surveillance and monitoring of cancer indicators in a pre-defined population. In particular, they provide data to pan-regional and pan-national cancer-information systems [1, 2] and also to international epidemiological studies on cancer incidence and mortality [3], and cancer survival [4, 5]. CRs need to be meticulous in collecting and verifying summary information on cancer cases occurring in their population-catchment areas. In coding this information, CRs make extensive use of harmonised classification schemes, such as the UICC TNM classification, which is an internationally accepted classification for staging solid tumours [6]. Tumour stage essentially describes the extent of the cancer in terms of growth and spread and is necessary for planning the most effective course of treatment. It is also important for estimating prognosis, as well as for evaluating the effectiveness of cancer-screening programmes.

TNM is a complex classification scheme however and is known to cause difficulties in clinical staging. Given the implication on patients, the observed deviation rates of 20% for clinical coding and 10% for pathological coding are considered very high [7]. Verification of the stage assigned to the cancer at diagnosis is therefore one of the most critical checks performed by CRs.

### TNM classification

The letters T, N, and M denote categories that describe respectively: the primary tumour size, the regional lymph node involvement, and distant metastatic spread. The values and meaning of T, N, M are dependent on cancer sites (topography) and morphology. For carcinoma breast cancer a value of T1 indicates a tumour dimension equal to or less than 2 cm whereas a value of T3 means a tumour size more than 5 cm; a value of N1 means that cancer cells have spread to one or more lymph nodes; and a value of M0 indicates that the cancer has not spread to other organs whereas a value of M1 indicates it has. For colorectal cancer, a value of T1 indicates the tumour has grown through the mucosa into the submucosa. A number of editions of TNM have been published and the latest edition published in 2017 is edition 8.

Cancer may be staged both clinically at diagnosis and also post-operatively following diagnosis from pathological examination of the excised primary tumour. This gives rise to the terms clinical TNM (cTNM or simply TNM) and pathological TNM (pTNM). Clinical stage generally plays the most important role for the first treatment course (e.g. neoadjuvant treatment), whereas pathology stage is used for planning additional treatment or to finalise the first course in a specific direction (e.g. type of adjuvant treatment).

The range of codes associated with each of the T, N, M categories are dependent not only on the TNM tumour site but also on the TNM edition itself. In order to simplify these category classifications, a more synthesised stage grouping structure is defined by one of the Roman numerals from 0 to IV, where: stage 0 indicates in situ cancer and is in the earliest stage of development; stage I signifies small, localised tumours; stage II and III signifies larger tumours with varying levels of infiltration to adjoining tissue or lymph nodes; and stage IV indicates metastasized tumours. Owing to the fact that some CRs have records of cancer cases stretching back a number of decades, they need to work simultaneously with different TNM editions (previous records are not normally mapped to later TNM editions for the reason that there may be no simple mapping).

### CR TNM-related tasks

Table 1 (after Martos et al. [8]), shows an example of TNM classification in relation to the permissible T, N, and M values for each of the recognised breast-cancer stages (TNM Edition 7). The references to “Any” (c.f. T and N codes under stages IIIC and IV) signifies any of the T, N codes referred to in this particular table. The relative laxity of this terminology provides an example of how a formal representation of a rule can avoid ambiguities. A precursory glance at this rule might lead the unwary reader to conclude that any value of T were allowed rather than any value in the specific set defined for breast cancer. Other TNM codes, in addition to topography and morphology depend on grade (e.g. bone and soft tissues) and also on age (thyroid gland).

**Table 1:**
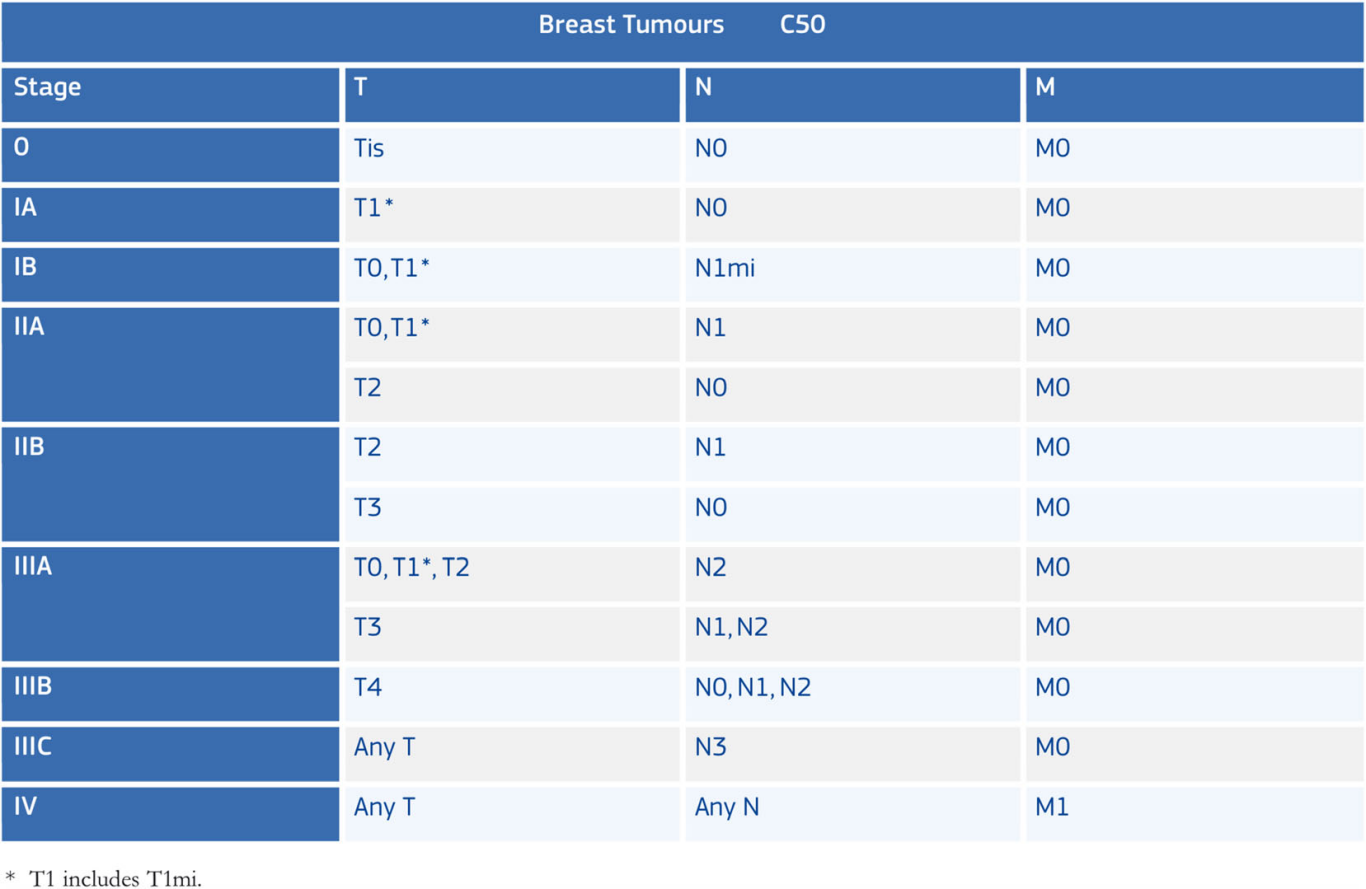
Permissible values and combinations of T, N, M, and stage codes (TNM edition 7) for breast cancers, after Martos et al. [8]

One of the primary tasks CRs have in relation to TNM is to check that the synthesised stage group accords with the T, N, and M category codes for the different TNM sites (of which there are approximately 55, depending on the TNM edition). Due to the different possible values within the TNM categories (especially the T and N categories) and the dependence of those values on anatomical site, tumour morphology, and on TNM edition, TNM is one of the most intricate validation checks of the data-cleaning process and few tools are available to support the operation. Apart from data-validation, CRs may also need to classify tumours based on the TNM parameters [9] as well as use TNM classification in the analysis of disease progression [10]. Having access to a single tool to address the various CR TNM-related needs would therefore be of great advantage.

### Ontologies

OWL-based ontologies are becoming common in the medical and biological sciences domains due to their ability to handle complex classification structures that do not necessarily have well defined hierarchical boundaries. Many of these ontologies may be found on Bioportal [11], a comprehensive repository of biomedical ontologies or on the Open Biological and Biomedical Ontology (OBO) Foundry [12] which aims to develop a set of interoperable ontologies that are logically and scientifically accurate for the biological sciences. Ontologies relevant to coding of medical and clinical terms include SNOMED [13], which is developed with the Web Ontology Language (OWL [14]). OpenGalen [15], and in reference to cancer, NCIthesaurus (NCIt [16]).

OWL ontologies provide three particular benefits. Firstly, they are based on description logic and offer automatic machine-reasoning capabilities allowing the possibility to draw logical inferences from the associated axioms. Such tools are particularly useful for validating data models and also for understanding relationships between entities in very large classification structures that may otherwise be overlooked. Secondly, they are able to describe relationships between entities in a formal way, thereby avoiding the ambiguities inherent in natural language. Lastly, they can build on each other in relatively straightforward ways if designed appropriately, which serves to aid reuse and semantic interoperation as well as allowing faster development times. One of the standard ways to accomplish this is via the import mechanism, whereby one ontology imports another and has immediate and direct access to the classes and properties of both ontologies. The importing ontology is then able to create extra axioms to define relationships between the classes of the imported ontologies similar to the manner described in the "ECR TNM-o v2 ontology structure" section.

It is however recognised that building ontologies is not a straightforward task [17] and care needs to be taken to ensure that an ontology is neither too specific to a given data domain as to result in a multiplication of ontologies for each individual task, nor too general so as not to be able to serve any application in particular. Efforts to reuse ontologies can be frustrated depending on the design and efficiency constraints of the individual ontologies [18]. Integrating and interfacing ontologies remains an active field of research [19].

### TNM-related ontologies

Most of the available standard biomedical ontologies have been developed in general either for clinical purposes or biomedical application/research and are geared primarily to those needs. For example, NCIt contains a controlled vocabulary for clinical care, translational and basic research, and public information and administrative activities. Whereas these resources are indispensable for ensuring correct semantic linkage between terminology systems and can support cross-domain inferences by that vocabulary linkage, they do not provide per se the automatic inference functionality in relation to specific sets of rules.

As far as the authors are aware, no comprehensive and formal TNM ontology exists for all tumour locations. As pointed out in [9], NCIt and SNOMED provide the general concept of tumour stage but do not contain the axioms for inferring it given a specific set of parameters and cannot be used for this purpose. Earlier work on ontologies for clinical staging addressed lung [20] and brain [21] tumours. Alfonse et al. [22] developed an ontology-based system for cancer diseases knowledge management for determining stage of cancer and subsequently the treatment regime for use by patients and physicians. The sites included lung, breast, and liver. Boeker et al. [7] developed a TNM ontology for deriving the correct values of the T, N, and M categories from specific pathology data. The ontology is comprehensive to the degree that it needs exact information of the tumour, including fine-grained information on the primary site of the cancer a well as the infiltration pattern. In virtue of the rich descriptive nature of the ontology to meet the purpose for which it was designed, the authors noted that the computational resources needed to classify the ontology were considerable and proposed further developing it as a system of modules for the different types of in situ tumours. At the time of writing only breast and colorectal tumours had been modelled. Moreover, the requirements of the clinical/pathological cancer-diagnosis processes are quite different from the downstream processes of validation of cancer-case records. An all-inclusive TNM-ontology is therefore likely to impose prohibitive processing times on current automatic reasoning algorithms for applications that need them, especially taking into account the requirement to model different TNM editions.

In contrast to the requirements of the clinical setting, population-based CRs deal for the most part with summary case information and do not need to incorporate all the specific information needed to derive stage from clinical/pathological examinations. A necessary part of the CR process is to check the validity of case records on the basis of the summary information provided and to ensure that the stage group provided is in accordance with the individual codes of the T, N, and M categories for a given cancer site specified in terms of ICD-O topography and morphology codes. ICD-O (International Classification of Diseases for oncology – currently in version 3.2, ICD-O-3 [23]) is a more descriptive classification scheme for cancer than ICD (International Classification of Diseases [24]); the main difference being that it separates morphology (describing the form/structure of the tumour) and behaviour (specifying the nature of the tumour – whether it is benign, in situ, malignant or uncertain/borderline) from topography (tumour location). An example of an ICD-O morphology code is M-8140/3, where the leading code “8140” specifies the tumour/cell type (in this case adenocarcinoma) and the trailing digit “3” specifies behaviour (e.g. malignant). In addition to the topography, morphology, and behaviour codes, ICO-O has a further one-digit code to describe histologic grading or differentiation, which is also required in the stage encoding of certain TNM topographic sites, such as bone. The more specific TNM ontologies developed for the clinical setting are thus not so well suited to the different needs of CRs with the requirements for TNM summary information comprehensive of all cancer sites specified in terms of ICD-O codes.

### TNM ontologies specific to CR needs

Massicano et al. [9] developed an ontology for the CR setting with the purpose of deriving stage from the TNM-edition 6 classification and limited the expressivity to $\mathrm{ALCI}$ (Attributive Concept Language with Complements and Inverse properties) allowing fast computational reasoning times. The ontology however was also not comprehensive of all cancer sites and did not include morphological information in terms of ICD-O-3 morphological codes and behaviour.

We previously developed a prototype data-validation tool [25] for CR data harmonised to a common data set [8] defined by the European Network of Cancer Registries (ENCR) and the European Commission’s Joint Research Centre (JRC). This data-validation tool included a TNM ontology (ENCR TNM-o) designed to incorporate morphology categories used by other components of the validation ontology. ENCR TNM-o was comprehensive of all sites (specified in terms of ICD-O-3 topographic codes and TNM edition 6) but was dependent on morphology only indirectly via the permissible ICD-O-3 morphology-topography combinations specified in the ENCR validation rules. The axioms were also specified in a different way from those developed in [9].

It was with the aim of creating a general-purpose TNM-stage ontology applicable to many of the TNM-related tasks in a CR that we redeveloped the TNM module of the ENCR data-validation tool incorporating aspects of the design described in [9] whilst including all the essential aspects required by the rigorous demands of the data validation checks.

This paper presents the unified TNM ontology (ENCR TNM-o v2) and shows from the design aspect how it is poised to serve a wide range of CR needs – namely in relation to knowledge management of the topography and morphology codes and their groupings; TNM codes, sites, stage groups and TNM editions; as well as in aiding and automating the various data-validation and error-correction processes.

## Implementation

### OWL class hierarchies used in the different CR TNM ontologies

**Fig. 1:**
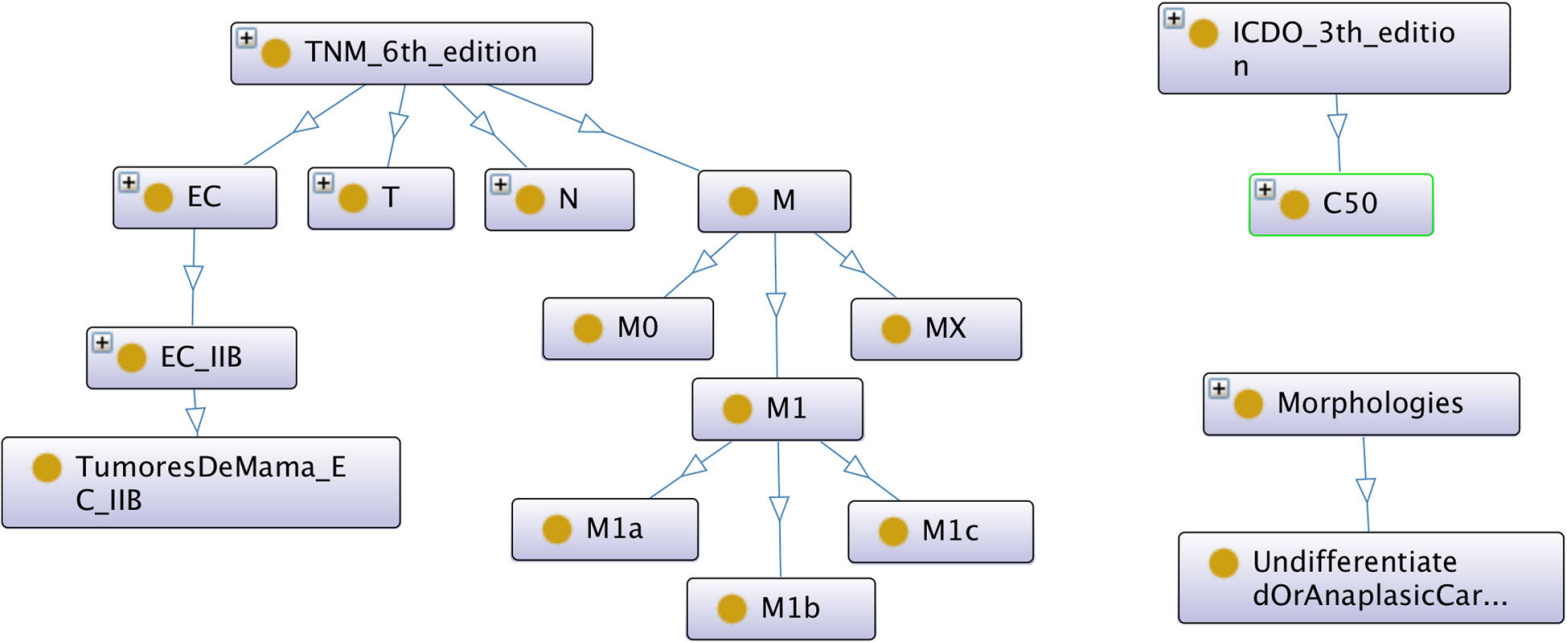
Class structure of the TNM ontology developed in [9]. Arrows point to subclasses and “+” signs in the top left corner of certain classes indicate the class contains more subclasses than those shown

**Fig. 2:**
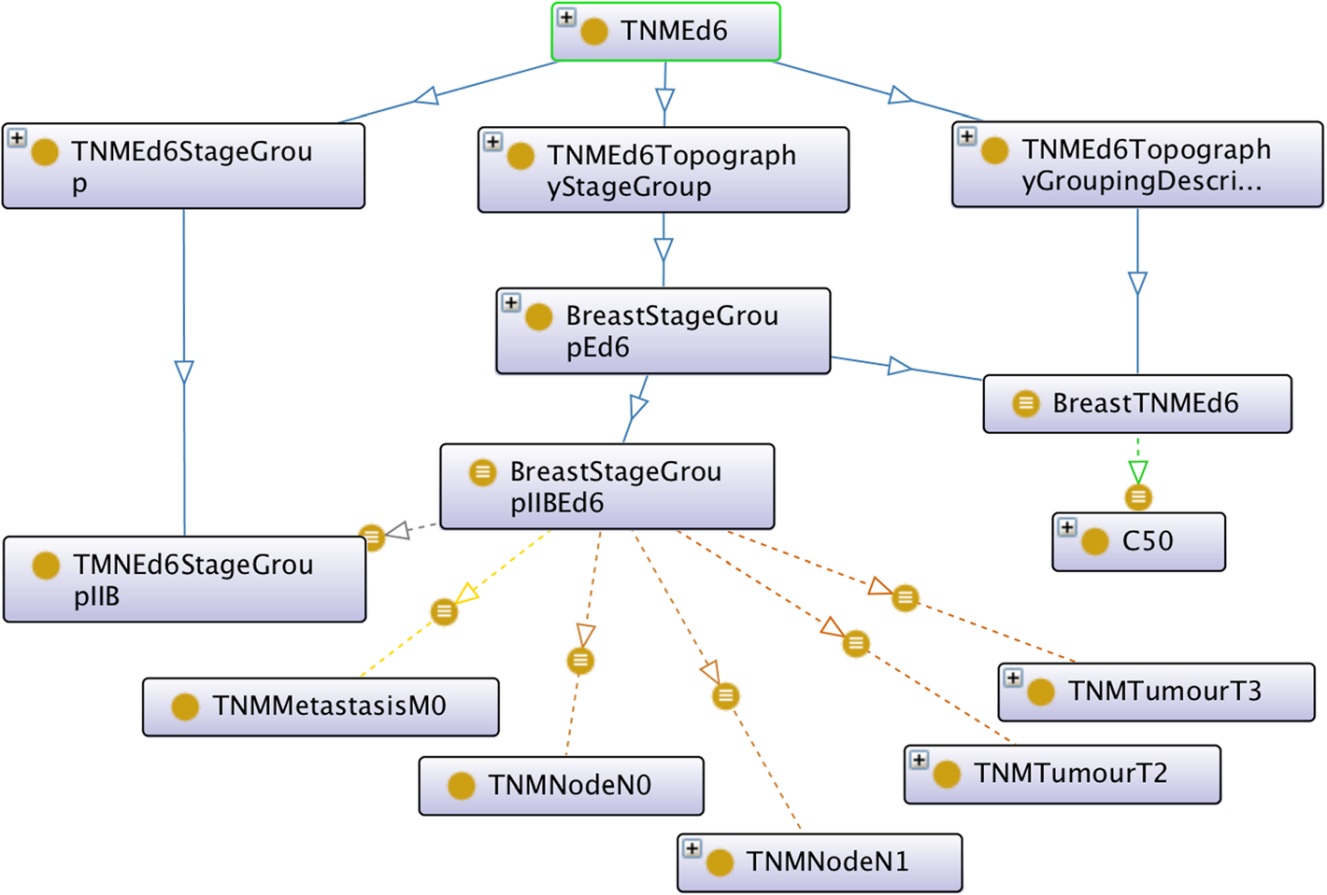
Class structure of the original ENCR TNM-o ontology used in the ENCR validation checks developed by the authors. Solid lines signify subclasses; broken lines signify object properties (with different colours representing different object properties); and brown circles touching the arrows denote equivalences

Figures 1 and 2 show the class hierarchy of the original two CR TNM ontologies (the one described in [9], and ENCR TNM-o respectively) expanded in part for stage IIB of the TNM site “breast”. Both ontologies classify stage groups according to the different TNM sites (e.g. breast stage IIB). An advantage of the ontology in Fig. 2 is that it is immediately apparent which values of the TNM parameters are possible within a given stage and TNM site and also which ICD-O-3 topography codes are associated with breast cancer. A disadvantage is the number of equivalent classes that are necessary (indicated by the small brown circles at the end of the arrows), which can lead to subtle types of error of unintended equivalences.

In view of the advantages accruing from the availability of having a single ontology for addressing the multiple needs of a CR, ENCR TNM-o was refactored to allow a complete separation of the major underlying validation components. This allows ENCR TNM-o v2 to be used in isolation and independently of the ENCR data-validation checks.

**Fig. 3:**
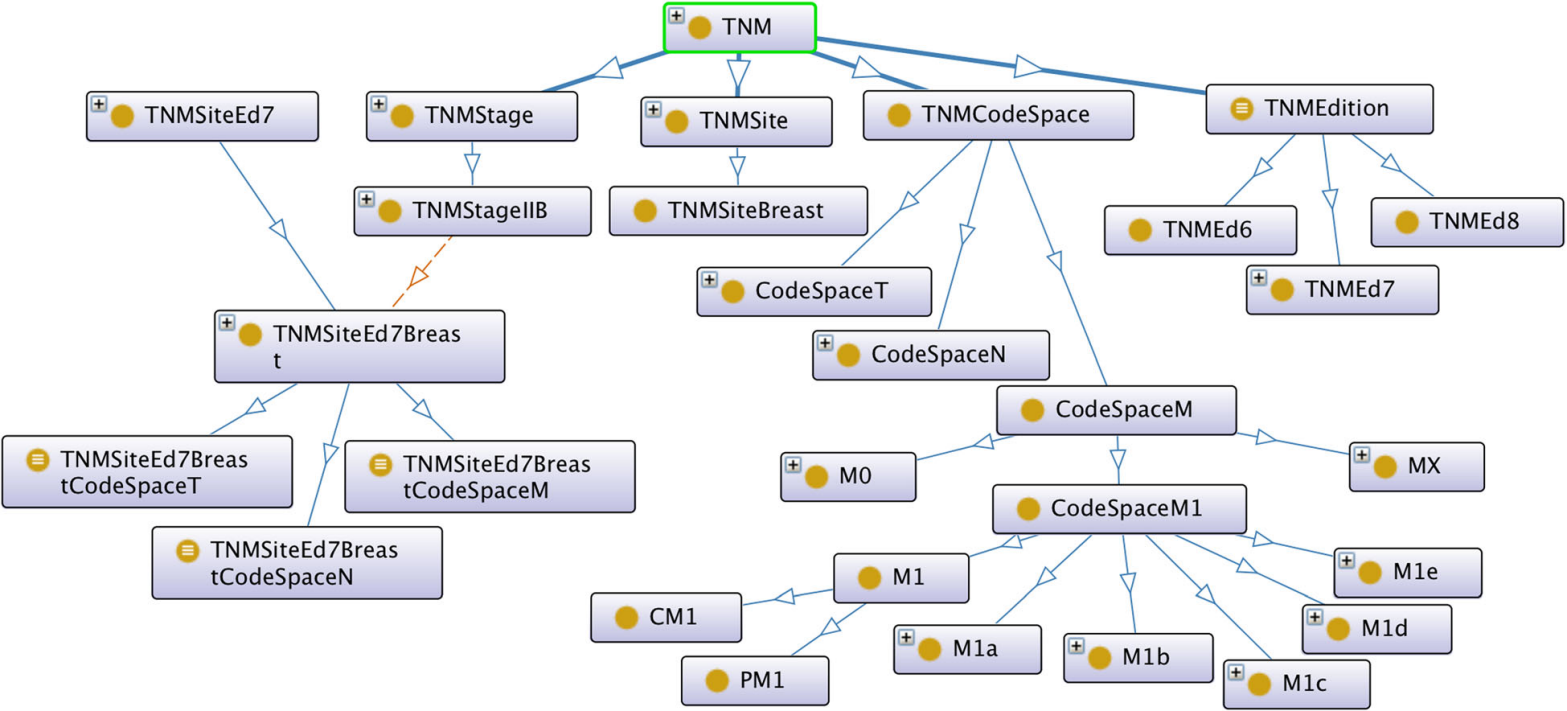
Class structure of ENCR TNM-o v2 showing the TNM-related associations. Solid lines signify subclasses; broken lines signify object properties

**Fig. 4:**
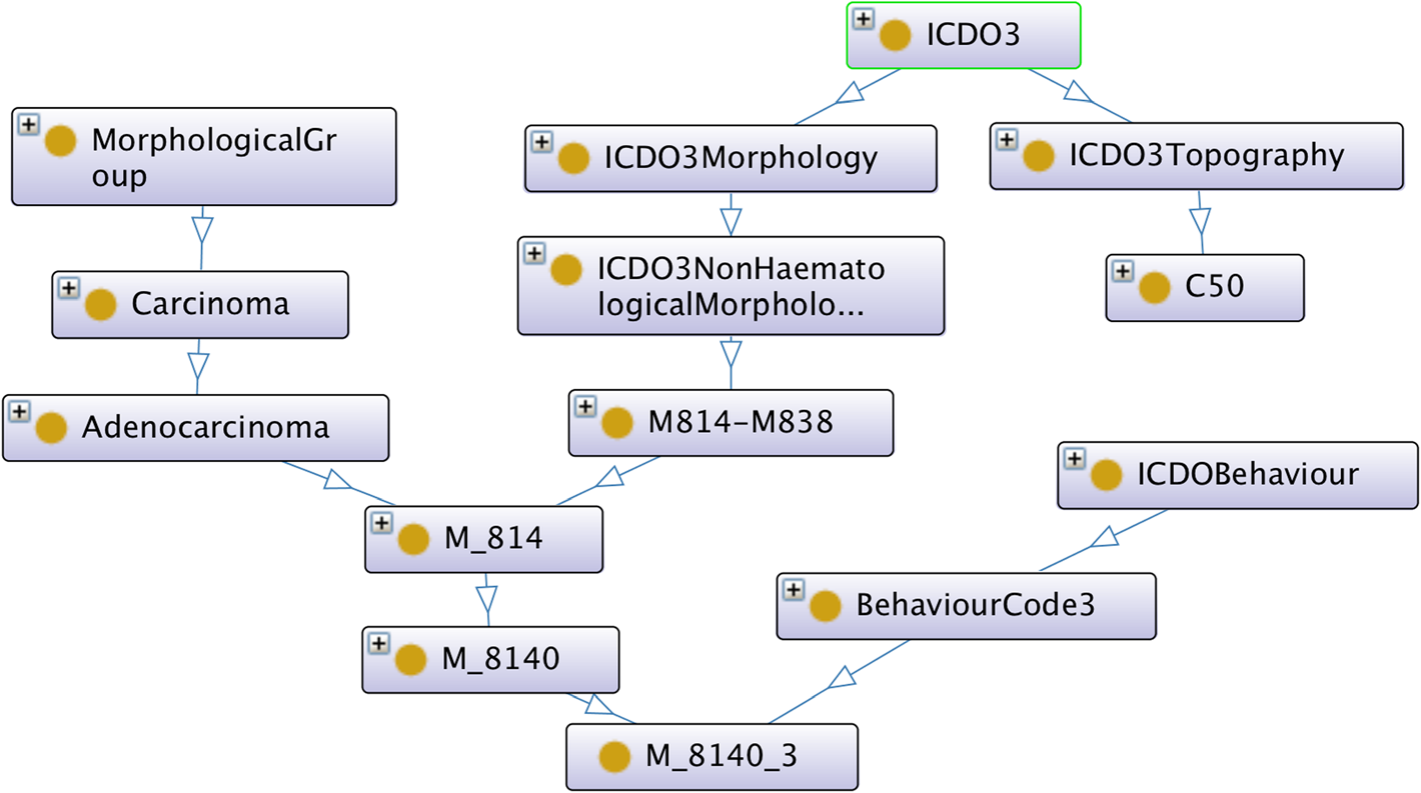
Class structure of ENCR TNM-o v2 showing the ICD-O-3 related associations

In order to achieve the unification of the two original TNM ontologies, the design of the axioms was aligned as far as possible with that of [9] but extended on the basis of the data-validation ontology to incorporate all TNM sites, all codes of the individual T, N, and M parameters, and all ICD-O-3 morphology codes grouped by morphology categorisation. Figures 3 and 4 show this alignment for ENCR TNM-o v2 (which is discussed further in the "Ontology design" section). Figure 3 illustrates the TNM-related classes, and Fig. 4, the ICD-O related classes.

In Fig. 3, the classes TNMStage and TNMStageIIB, correspond to the respective classes of EC and EC_IIB of Fig. 1, but there is a important distinction in the resultant subclass name (c.f. TNMSiteEd7Breast and TumoresDeMama_EC_IIB). We considered it important to decouple the TNM site (e.g. breast) from TNM stage (e.g. stage IIB) since the concept of stage is essentially independent of the specific cancer site. We also divided the TNM classes more comprehensively between a generic ontology and a TNM edition-specific ontology to avoid having to redefine all the TNM classes for each TNM specific edition. Furthermore, we introduced a TNMCodeSpace class to encapsulate the different permissible values for the T, N, and M parameters for the different cancer sites.

**Fig. 5:**
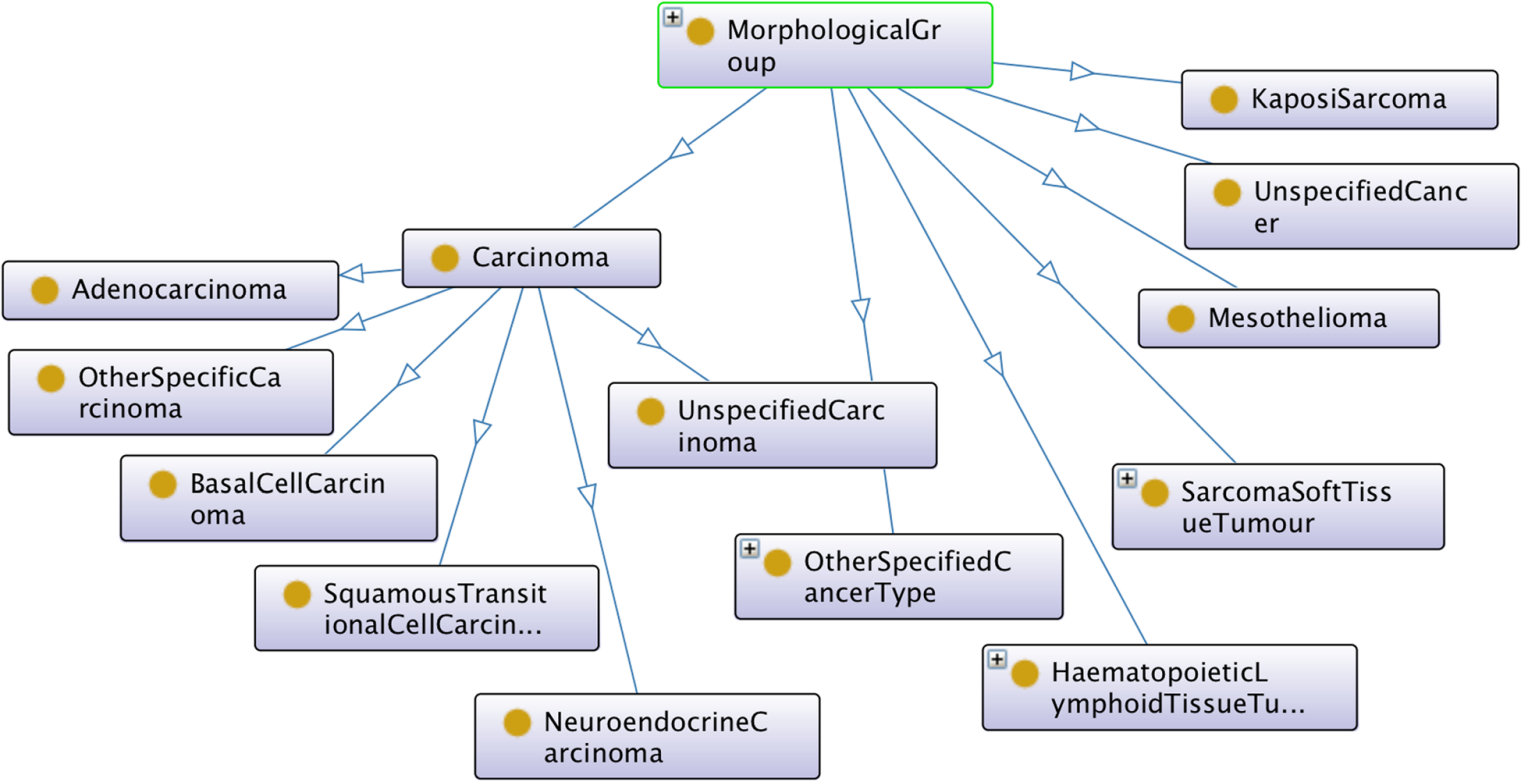
Class structure of MorphologicalGroup in ENCR TNM-o v2 showing some of the morphological categories expanded in part for the carcinoma class

Regarding the relation with ICD-O-3, all morphology codes have been defined and grouped under specific morphological categories in ENCR TNM-o v2, which are partly shown in Fig. 5. One example of the relationship between morphology code and morphology category is shown in Fig. 4 for the morphology code M_8140_3 and the adenocarcinoma morphology category. This is in contrast to Fig. 1, where only a descriptive morphological term is used (c.f. the UndifferentiatedOrAnaplasticCarcinoma class). ENCR TNM-o v2 also differentiates between pathological and clinical TNM. The resulting ontology is thus a more comprehensive model and more readily scalable to different TNM editions.

### ENCR TNM-o v2 ontology structure

ENCR TNM-o v2 draws on concepts that go beyond TNM and which serve other needs within the wider context of the work of CRs. Examples include the ICD-O-3 codes (broken down into their constituent parts, e.g. topography, morphology, behaviour codes, etc.) and the grouping of sets of morphology codes into relevant morphological categories (describing carcinomas, melanomas, sarcomas, etc.).

In order to provide a separation of these concerns and allow optimal reuse, ENCR TNM-o v2 is based on a modular design, in which the individual concerns or domains are encapsulated in separate ontologies. OWL ontologies (essentially files written in OWL) may import other OWL ontologies/files to build larger ontologies consisting of a number of separate ontologies. By modular design, we intend the separation of inherently different concerns into different abstractions, encapsulated in their own separate ontologies, which nevertheless can be integrated in a larger ontology and linked in an appropriate manner within that ontology whilst not interfering with their individual descriptions and/or axiomatic definitions.

**Fig. 6:**
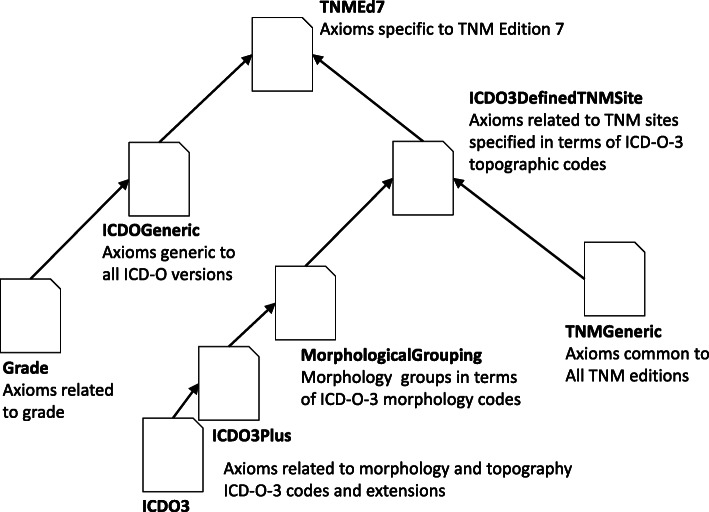
Structure of the ontology import tree. An ontology that points to another ontology is imported by the ontology pointed at. The structure is adaptable to any classification of codes and TNM edition; only the relevant ontology needs to be swapped out

The concept is illustrated in Fig. 6, which shows the import structure of ENCR TNM-o v2 whereby an ontology is imported by another in the direction of an arrowed line. An overview of some of the metrics associated with the constituent ontologies is provided in Table 2 where numbers are cumulative for ontologies which import others unless parenthesised when they show the ontology-specific numbers. Concerning the metrics: Class count refers to the number of distinct classes; SubClassOf refers to the number of SubClassOf axioms (through which a class is made a subclass of another named or unnamed class); Object property is the number of object properties; Equivalent classes is the number of equivalent or defined classes; GCI count refers to the number of general concept inclusions or SubClassOf axioms whose subclasses are complex class axioms; and Logical axiom count is the number of logical axioms (includes SubClassOf but not Class count).

**Table 2:** Overview of the individual ontologies shown in Fig. 6

	ICDO3 Plus	Morph Group	ICDO3 Defined TNMSite	TNM Generic	Grade	ICDO Generic	TNMEd7
Expressivity	ALC ^(D)^	ALCI^(D)^	ALCI^(D)^	ALC ^(D)^	AL	ALC	ALCI^(D)^
Logical axiom count	3744	4456 *(712)*	5344 *(386)*	502	5	44 *(39)*	6946 *(1558)*
Class count	2617	2649 *(32)*	2949 *(0)*	300	6	31 *(25)*	3256 *(276)*
SubclassOf	3738	4445 *(707)*	5241 *(386)*	410	5	27 *(22)*	6630 *(1362)*
GCI count	0	345	732 *(386)*	1	0	0	1555 *(823)*
Object property	3	4 *(1)*	16 *(0)*	12	1	5 *(4)*	21 *(0)*
Equivalent classes	1	1	3 *(0)*	2	0	9	203 *(191)*

The expressivity is also cumulative, where the meanings are: AL – Attributive Language or the basic description language; ALC – AL with complements (including full existential quantification and concept union); ALCI – ALC with inverse properties; The superscript D denotes datatype properties (used for specifying age in the axioms of TNM sites that require it, e.g. thyroid gland).

Consequently, the TNM axioms can be specified according to any TNM edition and to define an ontology of another TNM edition, only the axioms specific to that TNM edition have to be defined; the rest of the ontology structure remains the same. The structure is therefore adaptable and scalable to any particular edition of TNM. In a similar fashion, it is also possible to change the morphology code groupings in the MorphologyGrouping ontology without having necessarily to change all the associated TNM-related axioms.

There is nevertheless a significant number of classes within the edition-specific ontology to change (c.f. the bracketed numbers in the final column of Table 2). It is, however, a relatively straightforward task to make global replacements of version-dependent stings (e.g. TNMEd7 to TNMEd8) in an OWL file and once that is done, to tweak the individual classes where there are differences between the editions. Furthermore, once the edition-specific ontology has been finalised, there is thereafter no general need for changing it further. Whereas it could in principle be possible to define many of the stage-related TNM parameters in the generic TNM ontology (since many of them are identical between editions), it then becomes a more complicated maintenance task should a future TNM edition require changes to a rule that was common to all the previous editions (the common rule would then need to be removed from the TNM generic ontology and refactored in all the TNM edition-specific ontologies).

Once the ontology of a new TNM edition has been developed in this manner, it does require full testing, especially of the classification structures that have changed between editions. This is generally performed by passing a set of test records through the reasoner using the programme interface (described further in the "Results" section) and verifying the inferred stage is the same as that specified in each test record.

### Ontology design

OWL is based on the open-world assumption (OWA) which limits the inferences that can be made by any reasoning mechanism on statements known to be true – the philosophy being that there may be other information not yet known to the reasoner that may invalidate the inferences drawn.

In an OWL ontology, a reasoner can infer further classifications on the basis of information that is known and through which inferences can be made. OWL provides a number of mechanisms for imposing restrictions on the information available that allow such inferences to be made. One of the mechanisms relates to the “defined class” attribute. Defined classes essentially express equivalence. Defined classes are considered to contain a set of necessary and sufficient conditions that will make it automatically equivalent to any other class containing those same conditions. Thus in description logic, the axiom:

TNMSiteKidney ≡ ∃hasMorphology.Carcinoma ⊓ ∃hasTopography.C649

states an equivalence between the class TNMSiteKidney and the intersection of the object property hasMorphology having some carcinoma with the object property hasTopography having some ICD-O-3 topography code C64.9.

Another mechanism is via the general concept inclusion (GCI) construct [26] whereby an anonymous (or complex) class expression class is subclassed from an atomic class (in contrast to the more usual way of constructing classes using an OWL user interface such as Protégé [27]). This mechanism results in the subsumption by the atomic class of any class that contains the conditions specified in the complex class expression.

Thus, if instead of making the class TNMSiteKidney a subclass of a complex class expression such as:

TNMSiteKidney ⊑ ∃hasMorphology.Carcinoma ⊓ ∃hasTopography.C649

the complex class expression is made a subclass of TNMSiteKidney:

∃hasMorphology.Carcinoma ⊓ ∃hasTopography.C649 ⊑ TNMSiteKidney

the effect is that any class will be subsumed by TNMSiteKidney if it contains the intersection of the two classes:

∃hasMorphology.Carcinoma and ∃hasTopography.C649

Depending on the type of information one wishes to extract from an ontology, both subclassing constructs may be useful and it is worth noting that if both expressions are declared simultaneously, one has by definition [28] the equivalent class:

TNMSiteKidney ≡ ∃hasMorphology.Carcinoma ⊓ ∃hasTopography.C649

Using defined classes with complex class expressions however can lead to unintentional equivalence inferences in cases where identical expressions occur in two or more defined classes. Where there are many such complex expressions, it becomes difficult to ensure clashes do not occur. For this reason, the GCI approach was considered the most appropriate even though it tended to increase the number of axioms. GCIs are also known to cause performance issues [29, 30] but it was considered preferable in order to avoid potentially subtle inference errors.

Many of the equivalence axioms used in the data-validation ontology were consequently refactored. For example, the morphology category axiom:

Mesothelioma ≡ (M_9050 ⊔ M_9051 ⊔ M_9052 ⊔ M_9053 ⊔ M_9054 ⊔ M_9055)

was remodelled as six separate general class axioms, following the pattern:

∃hasMorphology.M_905X ⊑ Mesothelioma

where “X” signifies values between 0 and 5.

The number of axioms could be reduced in some instances by using three-digit morphology codes (e.g. M_905) and making the latter the superclasses of the four-digit codes, e.g.:

∃hasMorphology.M_905 ⊑ Mesothelioma

where

M_9050, M_9051, M_9052, M_9053, M_9054, M_9055 ⊑ M_905

Using this pattern, the data-validation TNM ontology could be more closely aligned with that of [9].

Encoding of TNM stage is performed on the basis of the various permissible codes ascribed to the individual T, N, and M categories. The codes ascribed to the T, N, and M categories are dependent on topography or site of the primary tumour as well as on the TNM edition. This introduces the notion of a symbol code space for each category, and was modelled in the ontology by a defined class for each TNM site specified by the TNM edition. Since the TNM sites have unique names, any clashes in the equivalence statements are avoided. For example the code space for T for the TNM site Breast in TNM edition 7 is specified by the intersection of the Breast TNMEd7 site with the union of all the associated T codes:

TNMSiteEd7Breast ⊓ ∃hasT.(CT0 ⊔ CT1 ⊔ CT1a ⊔ CT1b ⊔ CT1c ⊔ CT2 ⊔ CT3 ⊔ CT4 ⊔ CT4a ⊔ CT4b ⊔ CT4c ⊔ CT4d ⊔ CTX ⊔ PT0 ⊔ PT1 ⊔ PT1a ⊔ PT1b ⊔ PT1c ⊔ PT1mi ⊔ PT2 ⊔ PT3 ⊔ PT4 ⊔ PT4a ⊔ PT4b ⊔ PT4c ⊔ PT4d ⊔ PTX ⊔ PTis) (1)

Where the classes prefixed by the letter “C” denote clinical T and those prefixed by the letter “P”, pathological T. Any T code outside this code space is not recognised for this particular TNM site. Also in this axiom, the class TNMSiteEd7Breast is the superclass of the intersection of the TNM generic name of the same site and the object property of hasTNMEdition acting on the class TNMEd7:

TNMSiteBreast ⊓ ∃hasTNMEdition.TNMEd7 ⊑ TNMSiteEd7Breast (2)

Finally, the TNM generic class for the TNM topographic site “Breast” is the superclass of the intersection of the object properties related to the ICD-O-3 topographic code C50 and the morphology category denoted by the Carcinoma class which itself consists of object properties related to a number of ICD-O-3 morphology codes:

∃hasTopography.C50 ⊓ ∃hasMorphology.Carcinoma ⊑ TNMSiteBreast

where the morphology category Carcinoma is the superclass of the morphology subcategory Adenocarcinoma.

Adenocarcinoma ⊑ Carcinoma

which is described in terms of specific ICD-O-3 morphology codes, one example being:

∃hasMorphology.M_850 ⊑ ∃hasMorphology.Adenocarcinoma

These aspects were not modelled in the ontology of [9]; also, instead of modelling stage as the intersection of the TNM category classes and topography class as in the example below for stage 0 breast cancer:

C50 ⊓ Tis ⊓ N0 ⊓ M0 ⊑ BreastCancer_CS_0

we preferred to represent a general class of stage 0 as an intersection of object properties (of T, N, and M) with the class TNMEd7Breast defined in axiom (2) and an object property of hasBehaviour with BehaviourCode2 (corresponding to in situ tumours):

TNMSiteEd7Breast ⊓ ∃hasBehaviour.BehavioutCode2 ⊓ ∃hasT.Tis ⊓ ∃hasN.N0 ⊓ ∃hasM.M0 ⊑ TNMStage0

Defining the axioms in this way reduces the need to create a separate class for each combination of stage group and TNM site and also allows the conceptually different classes of topography, T, N, and M to be declared disjoint.

Another aspect we modelled in ENCR TNM-o v2 was the concept of code-spaces for T, N, and M encapsulating all the respective codes for a given TNM cancer site. The permissible sets of codes are in general different for different cancer sites and this feature was not modelled in the ontology of [9], where for instance the axiom for stage IIIC breast cancer is:

C50 ⊓ N3 ⊓ M0 ⊑ BreastCancer_CS_IIIC

This axiom is entirely independent of T and would miss any associated data-validation errors. In ENCR TNM-o v2, the same class is modelled as:

TNMSiteEd7BreastCodeSpaceT ⊓ ∃hasN.N3 ⊓ ∃hasM.M0 ⊓ ∃hasBehaviour.BehaviourCode3 ⊑ TNMStageIIIC

where the first term is provided by axiom (1), in line with the set of T category values for breast cancers derived from Table 1.

By extending these axioms for the entire set of TNM cancer sites, the ontology is able to provide a comprehensive representation of the TNM tables such as those shown in Table 1. Moreover, defining the axioms in this way using general concept inclusions after the manner proposed in [9] provides the means of automatically deriving stage from knowledge of the parameters on which it depends.

#### Subsumption of classes

As a consequence of this design, an input record specifying an object property of hasMorphology with morphology code subclassed under M_850 will be subsumed under:

∃hasMorphology.Carcinoma

If the input record were also to specify an object property of hasTopography with a topography code in the class hierarchy of C50, this together with the morphological designation will be subsumed under TNMSiteBreast. TNMSiteBreast together with an object property of hasTNMEdition of TNMEd7, will in turn be subsumed under TNMSiteEd7Breast.

**Fig. 7:**
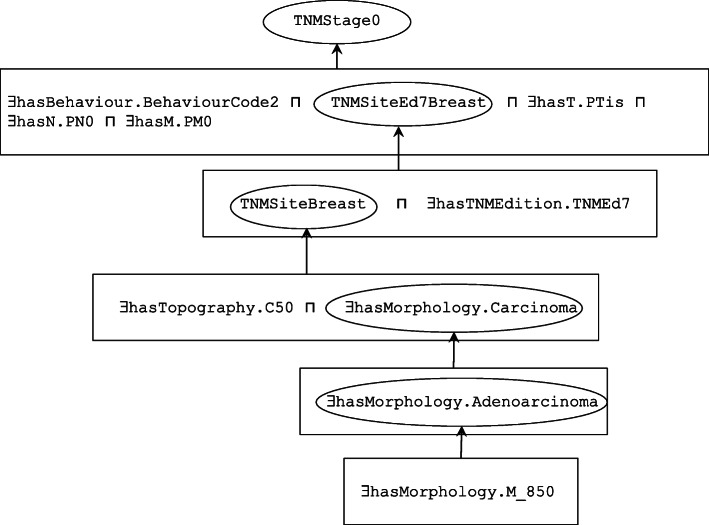
Subsumption schema of the GCI axioms in which the boxed axioms are subsumed by the circled classes

Finally, if the input record specified the object properties with the corresponding correct T, N, and M codes (in this case PTis, CN0 or PN0, and CM0 or PM0), the whole input record will be subsumed under TNMStage0. The subsumption schema for the breast cancer example just described is illustrated in Fig. 7 where the boxed axioms are subsumed by the circled classes to provide the final subsumption under TNM stage0.

The ontology takes into full consideration the ICD-O-3 morphology codes which are themselves classified according to the categories of malignant neoplasms specified in Table 25 of ICD-O-3 first revision [23] and adapted from [31]. The axioms also provide via the generic TNM classes a scalable architecture that minimises duplication of classes between different TNM editions. The design therefore provides a comprehensive basis of a general-purpose TNM ontology that can be useful for serving the various TNM-relate tasks within a CR.

#### Comparison of metrics of TNM ontologies

The expressivity of ENCR TNM-o v2 is nominally ALCIQ^(D)^ (ALCI with qualified cardinality restrictions) but the qualified cardinality restriction arises solely from one axiom in one of the imported ontologies and is not used explicitly within the TNM ontology; thus the expressivity can be considered as ALCI^(D)^ – the same level of expressivity as the ontology developed in [9]. Table 3 shows a comparison between the different ontologies using the same metrics as those described in the "ECR TNM-o v2 ontology structure" section.

**Table 3:** Comparison of axiom and class counts between the CR-related TNM ontologies. The metrics of the ontology of [9] were taken from the ontologies directly downloaded from: https://github.com/djogopatrao/tnm_ontology/tree/master/ontologies

	TNM ontology after Massicano et al.	TNM module of ENCR validation ontology	ENCR TNM-o v2
Expressivity	ALCI^(D)^	ALCI^(D)^	ALCI^(D)^
Logical axiom count	2397	5885	6946
Class count	583	4102	3256
SubclassOf	1143	2891	6630
GCI count	566	12	1555
Object Property	1	23	21
Equivalent classes	1	2836	203

## Results

The user has two ways of interfacing with ENCR TNM-o v2; either through the Protégé user interface or through a dedicated frontend Java programme that itself interfaces to the ontology via the OWL-API. The Protégé application is particularly useful for quickly ascertaining all the information pertaining to a given TNM cancer site (topography codes, morphology codes, T, N, and M parameter codes and their relation to the various allowed TNM stages), as well as for deriving stage information for a given set of cancer-case inputs. In contrast, the programme interface is more convenient for data validation purposes when dealing with the many tens and hundreds of thousands of records held within a CR. In this case, the TNM ontology is effectively used as a record validator and there is no need to add the entire CR data set to it in one go, which would otherwise affect the efficiency of the automatic reasoning due to the greatly increased number of axioms. Using the programme, the input data file can be ingested in stages with each stage adding a block of records transcribed in axiomatic form temporarily to the ontology for validation before being removed prior to the addition of the next block of records.

The Java programme can itself run in two modes – with and without reasoning. The ontology’s asserted axioms can be used directly to derive all the information needed without having to resort to the automatic reasoner and this programme mode was developed in order to compare computational times. The code in this case made extensive use of the OWL API [32] methods and pattern matching on the axiom strings to determine the correct parameter list associated with the input stage group and the TNM site derived from the input topography and morphology codes.

Using the Protégé user interface, input records can be inserted either as classes or individuals or as DL statements. The results from the reasoner are the same – there are no axioms that provide added functionality to motivate the need for individuals.

The results from some of the rudimentary benchmarking tests executed on an Apple MacBook Pro, processor 3 Ghz Intel Core i7 running macOS High Sierra v. 10.13.6 were as follows:

1. Using the Protégé tool (Protégé v.5.2.0 with the FaCT++ reasoner v.1.6.5), the time taken for the reasoner to classify the ontology and derive the stage group for a test case was approximately 4 s. This compares similarly with the TNM module of the earlier CR data validation tool but approximately twice the time for the ontology of [9] (which has about a third the number of axioms). Once the reasoner had loaded, the results from records input as DL statements were almost instantaneous;
2. Using the Java programme interface, the time taken for the programme to complete with an input file of 179 records once the ontologies had been imported was 7 s without reasoning and 15 s with reasoning (not including the one-off reasoner loading time) – neither of the Java code modalities were optimised. The records tested at least three scenarios for each TNM site: (i) parameters of input record specified correctly to enable verification of stage group with input value; (ii) one or more parameters specified incorrectly for the input stage group; (iii) an input value of stage group not recognised in the valid list of stage groups for the TNM site.

Besides the functionality to validate stage information of batch input records, the ontology provides a number of knowledge-management features that would find immediate application in the work of CRs. Some examples are provided in the following section.

### Information readily derived from the ontology

Information requirements of CRs include the need: (i) to ascertain the stage group for a given set of input parameters; (ii) to know all the possible stage groups for a given cancer site; and (iii) to know the individual morphology codes comprising a morphology category and, conversely, (iv) to know to which morphology group an individual morphology code belongs.

#### Determination of stage group from T, N, M parameters

**Fig. 8:**
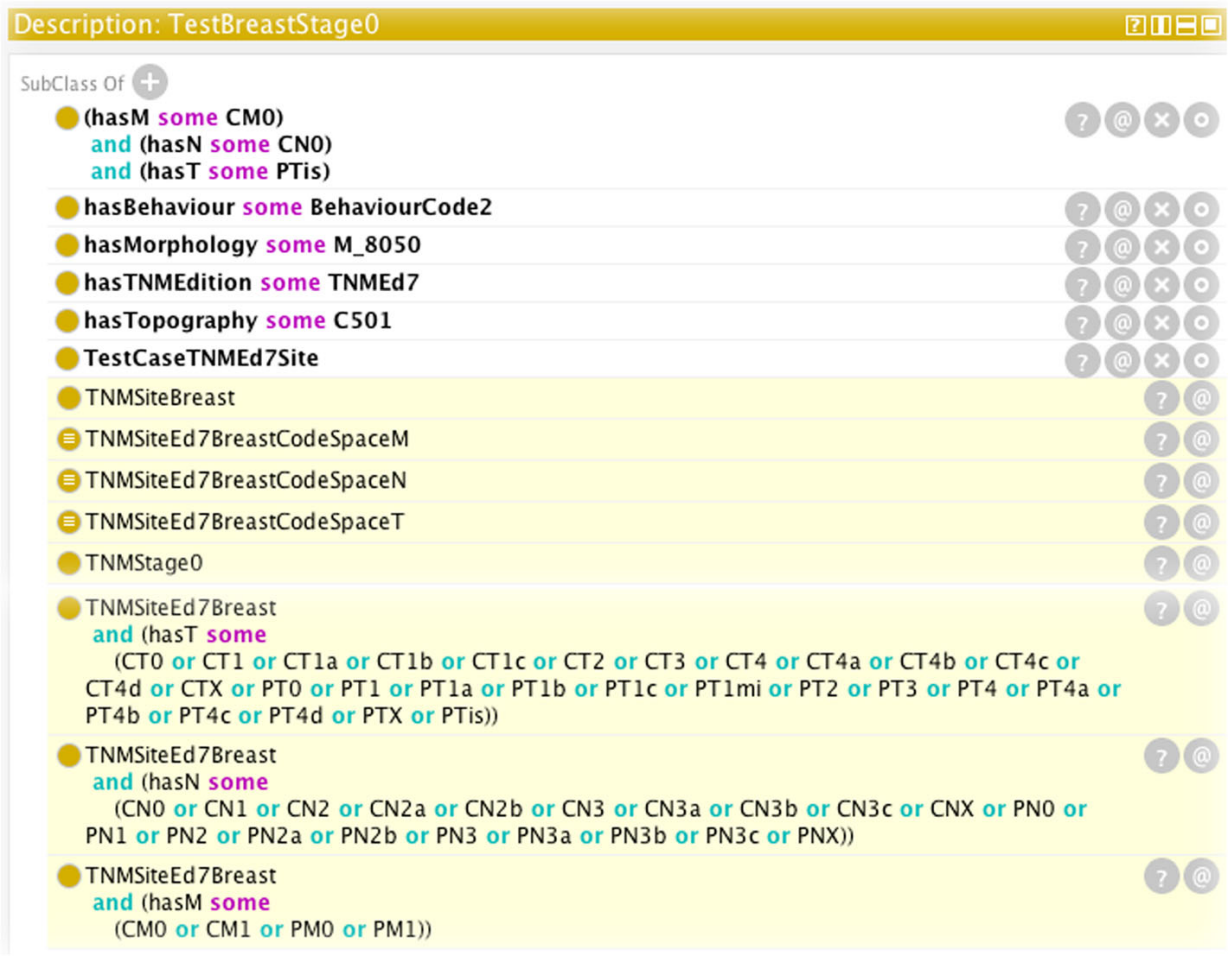
Result of the reasoning process for the input values provided in the top part of the figure with white background. The yellow highlighted text contains the values returned by the reasoner. The P/C prefixes of the T, N, M parameters refer to pathological TNM and clinical TNM respectively

As an example of the ontology’s ability to derive stage from a given set of parameters, Fig. 8 is a screen shot of the Protégé user interface showing the results inferred by the reasoner after having been passed a set of input parameters. The non-highlighted text indicates the input parameters (T, N, M, values, TNM edition, morphology, behaviour, and topography); the highlighted yellow background shows information inferred by the reasoner. On the basis of the subsumption model illustrated in the "Subsumption of classes" section, the reasoner has correctly ascertained the TNM site as Breast for the given ICD-O-3 topography code C501 and ICD-O-3 morphology code 8050/2 and also correctly determined the stage group as 0. Moreover, it has transcribed the code-space axioms associated with the TNM site from which it can be verified that the parameter values are within the permissible sets of values.

#### Determination of stage group values of a given TNM site

**Fig. 9:**
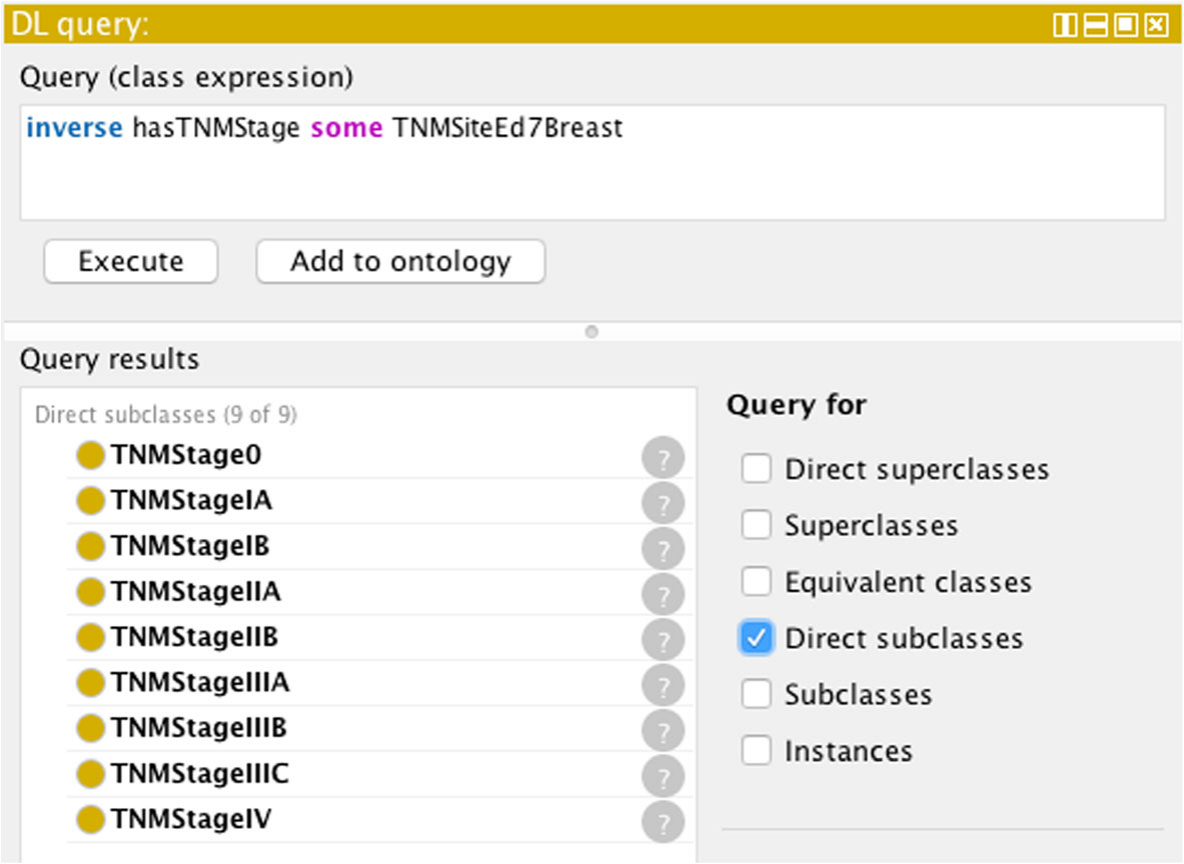
Result of a DL query by the class expression shown in the top part of the figure. The query returns all the permissible stage group values for the TNM edition 7 defined site corresponding to breast (c.f. Table 1)

The ontology is able simply from the specified inverse properties to return the list of permissible stage groups from a DL query on the cancer site – c.f. Figure 9 for the TNM (edition 7) site Breast:

#### Determination of morphology codes from a morphology category

**Fig. 10:**
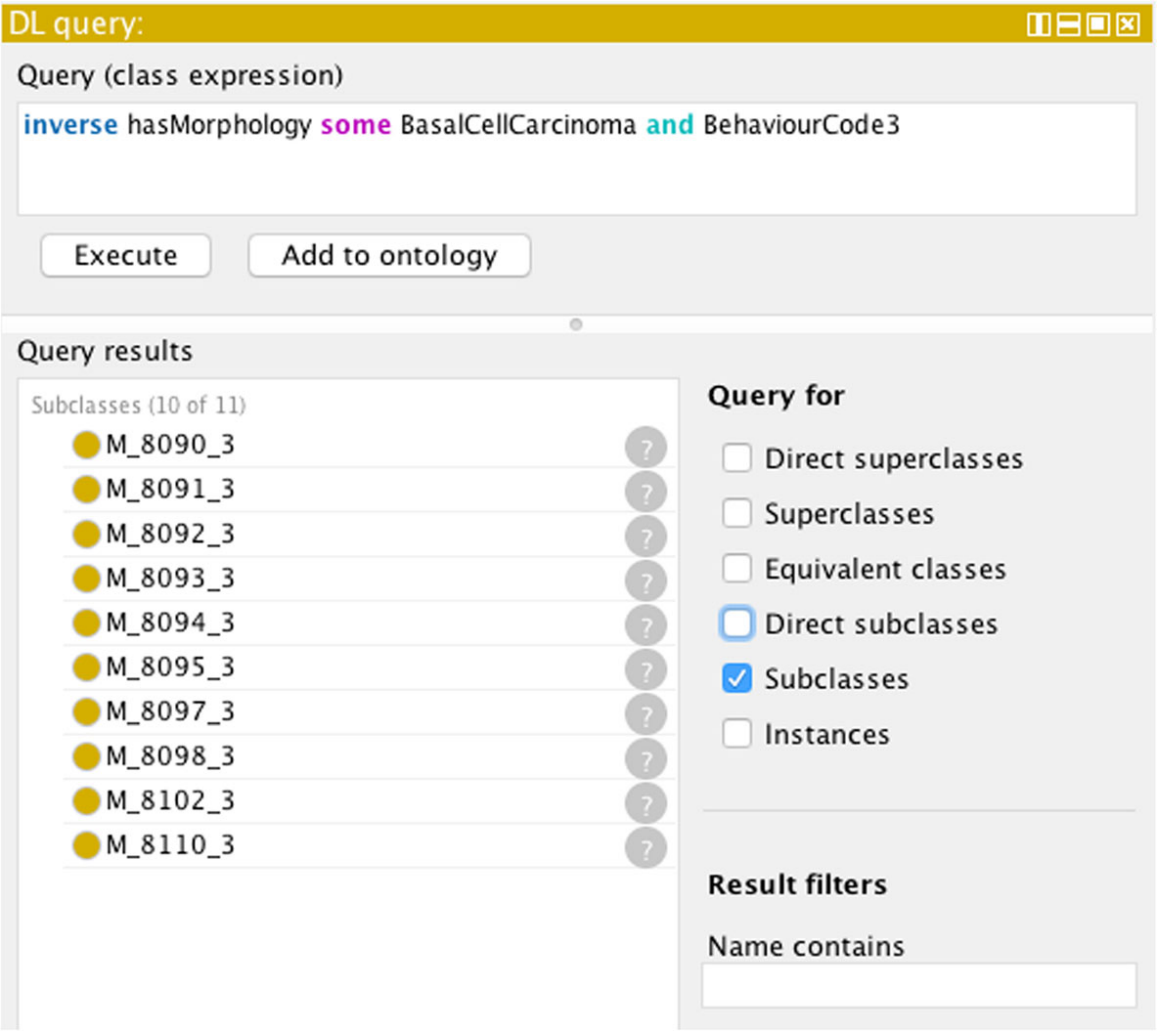
Result of a DL query returning the ICD-O-3 morphological codes associated with the morphological category basal cell carcinoma for morphologies (with behaviour code 3)

Figure 10 shows an example of how all the morphology codes (with behaviour 3) can be retrieved associated with basal cell carcinoma morphology category:

#### Determination of morphology category from a morphology code

**Fig. 11:**
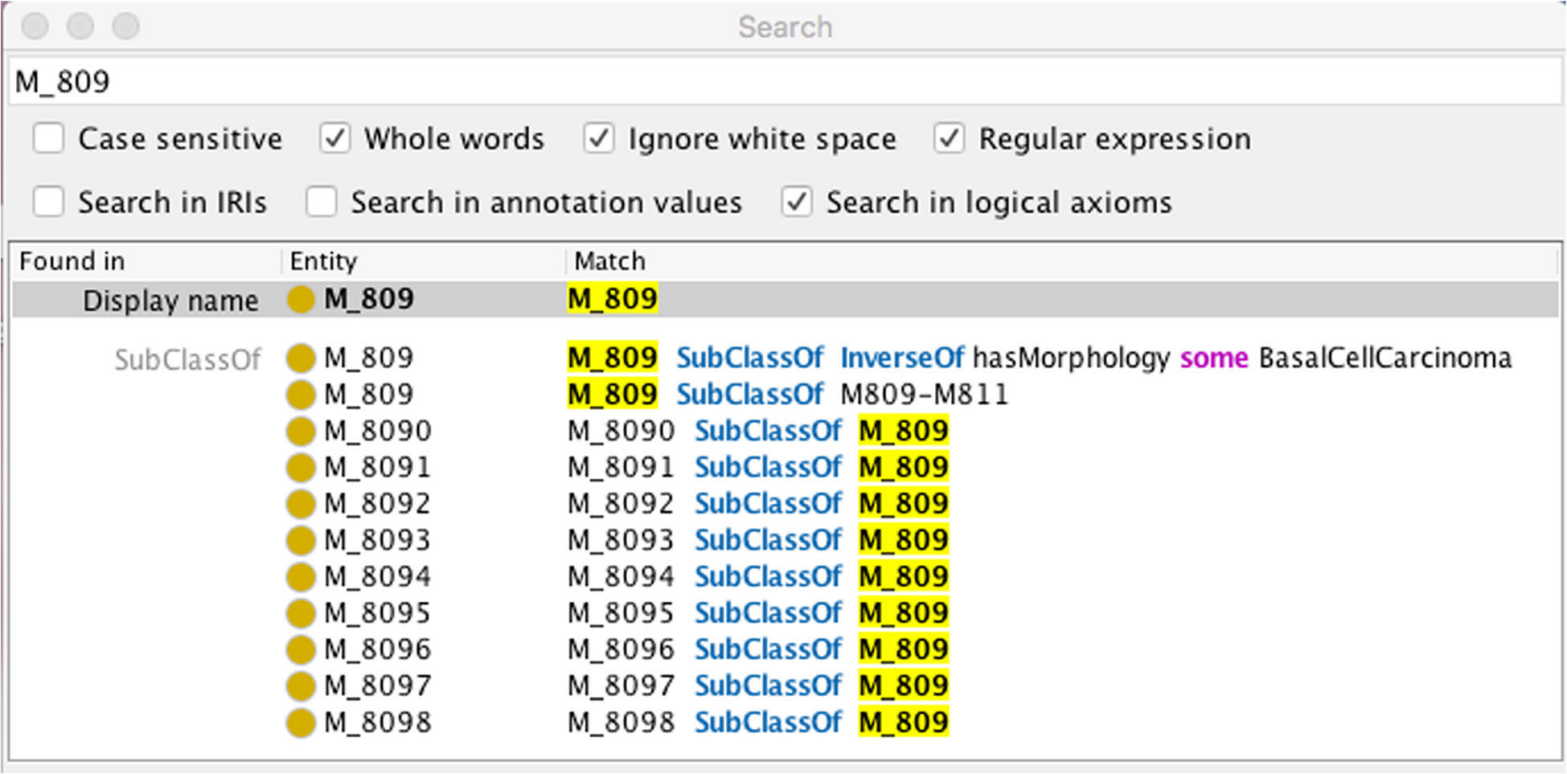
Result of a search on the asserted axioms for the ICD-O-3 morphology code M_809, which show that the morphology code is associated with the basal cell carcinoma morphology category

The converse case of determining morphology category for a given morphology code is equally straightforward using Protégé’s search function on the asserted axioms. Figure 11 shows that the specified morphology code M_809 belongs to the basal cell carcinoma morphology category.

The examples given here are not intended to provide a systematic overview of all the different possible types of scenarios but serve only to show how the ontology is able to meet the different types of information needs introduced at the beginning of the section. They are however representative of how the information is described in the similarly structured axioms for each TNM cancer site.

### Automatic error correction

**Fig. 12:**
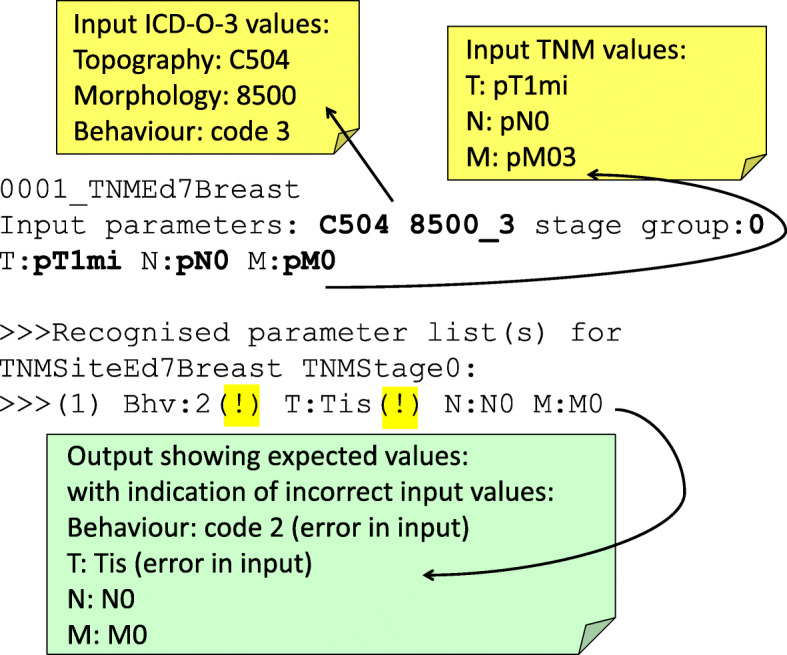
Programme output of a test record containing an error in the value of one of the input TNM parameters (T) and an error in the behaviour code. The input values are in bold font. The output follows the chevron symbols, and errors found in the input are indicated by an exclamation mark (highlighted) next to the expected values. This is an example of a case where automatic error correction could be made

The ontology provides the complete set of knowledge to be able to correct errors or at least to propose corrections. Figure 12 shows the output from the Java programme for a test record containing an error in the code assigned to the TNM “T” category, and value of behaviour. The expected values (c.f. Table 1, and also as inferred from the associated axiom for stage 0 breast cancer in Fig. 7) are provided in the programme output following the chevron symbols and where highlighted exclamation marks indicate incorrect values in the input record (which is indicated in bold text).

Using the knowledge contained within the ontology via the OWL-API methods, it is a relatively straightforward task to ascertain which of the codes has been incorrectly specified and in instances where the correct value(s) are uniquely ascertained (such as in the example given), the code could in principle provide automatic correction. In the current version of the code it is assumed that the stage group has been specified correctly and the error is in the other parameter values. The algorithm could be further developed to check for recognised stage group value on the assumption that all the parameter values had been specified correctly – this is in effect what is done by the code with the reasoner activated.

## Discussion

ENCR TNM-o v2 was developed with the aim of providing a multipurpose TNM tool to aid the TNM-related tasks confronting CRs. The intention was to unify an approach towards a common ontology and seek convergence of different ontology designs developed for different purposes.

The resulting ontology provides many different types of functionality that would predispose it to wide application in the work of CRs. Moreover, it addresses the needs for which previous CR-related TNM ontologies have been developed and could be integrated into those applications without undue difficulty.

The advantages of such a tool over traditional validation software accrue directly from the functionality provided by ontologies. Arguably the most important aspect concerns the transcription of rules expressed in natural language into ones that have formal representation (e.g, description logic). Formal representation removes much of the ambiguity that can otherwise arise. Moreover, ENCR TNM-o v2 incorporates the whole set of T, N, and M parameters and stage codes for all the cancer sites in the manner discussed in the "ENCR TNM-o v2 ontology structure" section and therefore provides a comprehensive knowledge base for CR TNM validation-checking software, which can be queried for the types of information described in the "Information readily derived from the ontology" section. In addition, OWL ontologies have a unique IRI that can help with version control. Circulation of different versions of application software can be difficult to control with downloadable software and can impact on devolved data-harmonisation processes. The latter require the assurance that the same version of validation-checking software has been used by all the associated data providers. Having one definitive access point to the maintained set of axioms can help ensure this.

Whereas the reasoning speeds reported in the "Results" section is not likely to pose any problem in the majority of applications, it may impact on requirements to validate large data files (in excess of several tens of thousands of records) in real time. Even in those specific cases reasoning speed is not likely to present an insurmountable hurdle – the validation process renders itself amenable to execution in background mode; it is not necessary that results are available immediately since the output log file describes in detail all the inconsistencies/errors found during the checks. In addition, processing of large CR data files is readily parallelisable simply by breaking the CR data files up into a set of smaller files. Gains in speed could also be achieved by optimising the code and axiom constructs as well as using a more performant processor. It should also be borne in mind that a substantial number of cancer-case records have no associated TNM data and therefore do not need to be passed to the TNM validation check, thereby reducing the overall size of the data file. Furthermore, with a dedicated programme interface, it is not necessary to have to rely on the automatic reasoner alone since the asserted axioms model the complete set of validation rules. Thus any errors can be tested via conditional statements within the code that interfaces to the ontology via the OWL-API. Doing this however results in greater coding effort and consequently in increased code-maintenance costs. As an indication of the latter, the code specific to the TNM stage group validation could be written with less than 300 lines using the automatic reasoner whereas without it, approximately 1000 lines of code were needed. Notwithstanding this fact, the coding effort and maintenance overheads are very much reduced to what they would otherwise be developing dedicated standalone software.

The foregoing discussion highlights another notable advantage of an ontology – its ability to handle the logic and axiomatic knowledge of a particular domain results in considerably less effort than in developing and maintaining traditional software applications/tools to do the same job. By confining the intelligence to the ontology, any interfacing software can be kept much lighter and more basic. Ontologies however need to be designed carefully to handle the delicate balance between a number of competing demands and the optimal design pattern is not necessarily immediately apparent. Ensuring subsumption only with class-specific (TBox) axioms under OWL’s OWA forces certain design constraints using either GCIs or equivalent classes. The latter can lead to subtle unintended equivalences that are difficult to control, especially when used in complex expression containing disjunctions. It is for this reason that ENCR TNM-o v2 used the GCI construct in preference to equivalent classes. Using SWRL (Semantic Web Rule Language) is a further approach that can be used but can lead to decidability and interoperability problems [33].

The axioms in ENCR TNM-o v2 do not check for errors such as the input of more than one topography or morphology code. Whereas checks of this kind could be modelled using individuals and qualified cardinality they would only unnecessarily affect performance further. Input errors like these are unlikely and generally immediately noticeable, at least for ad hoc checks added manually using the Protégé interface, due to the short record input (c.f. the un-highlighted text in the top five statements of Fig. 6). Moreover, with the programme interface used for the data-validation batch record checks, such errors are trapped using pre-processing code that automatically verifies the conformity of the input records to a standard record format template prior to inserting them into the ontology.

The next steps will be to benchmark the ontology and the interfacing software code with the existing production-level validation software (JRC-ENCR QCS [34]). Once the concept behind the ontology has been proven in the field, it will be further developed to incorporate all the other TNM editions concurrently in use within CRs. It will also replace the existing TNM module in the data-validation ontology application described in [25].

## Conclusion

The TNM ontology developed in this work provides a multi-purpose tool for TNM-related tasks in a CR. It is scalable for different editions of TNM. Besides offering a quick way of checking validity of cancer case stage information on the fly, it is adaptable to many uses, either as a standalone TNM module or as a component in applications of wider focus, such as in time analyses of disease courses or in full data validation/quality control. It provides a knowledge-management tool for modelling and retrieval of the relationships between hundreds of inter-dependent codes and offers reasoning capabilities for understanding the inconsistencies arising from the combination of these codes. With the added functionality provided by the interfacing software it is even possible to offer a parameterised means of correcting errors in certain instances.

The grounds for motivating the uptake of the ontology hinges amongst other things on an ontology’s ability to:

1. formalise the data-checking rules regarding TNM and remove the ambiguity of rules written in natural language;
2. incorporate comprehensive knowledge in terms of stage according to the parameter inputs of topography, morphology, grade, etc.;
3. provide the definitive knowledge base for any CR TNM validation-checking software, allowing the latter to be developed without the need to reformulate the rules and thereby risk introducing rule-based errors;
4. simplify the programming effort and costs otherwise required, in addition to the subsequent maintenance costs;
5. provide a sole reference point, critical for version control and synchronisation amongst applications that use it;
6. provide a standalone application in its own right using the readily available freeware Protégé user interface, for knowledge management purposes and for running ad hoc DL queries.

The endeavour towards ensuring a single, unified TNM CR ontology merits the effort involved. Its realisation will avoid the multiplicity of task-specific applications that lead to higher software maintenance costs and different metadata constructs for shared concepts, the inefficiency and inconveniences of which only propagate back to the user. This ontology hopefully provides an important step forward towards this goal.

## Availability and requirements

Project name: ENCR TNM ontology.

http://data.europa.eu/89h/9fa603ff-a118-41f3-82a2-bf8d4f0d7ea3

Operating system(s): Platform independent.

Programming language: Web Ontology Language (OWL).

Other requirements: Ontology editor (e.g. Protégé Desktop v.5.2.0).

License: BSD 2-clause licence (Protégé).

## Data Availability

The datasets generated during the current study are available in the Joint Research Centre Data Catalogue repository: http://data.europa.eu/89h/9fa603ff-a118-41f3-82a2-bf8d4f0d7ea3

## Abbreviations

ALCI(D), ALCIQ(D): expressivities of description logic
API: Application-programming interface
CR: Cancer registry
DL: Description logic
EC: European Commission
ECIS: European cancer information system
ENCR: European Network of Cancer Registries
EU: European Union
GCI: General concept inclusion
ICD: International Classification of Diseases (currently in 11th revision)
ICD-O: International Classification of Diseases for oncology (currently in 3rd edition)
IRI: International resource identifier
JRC: Joint Research Centre
NCIt: North American national cancer institute thesaurus
OBO: Open Biological and Biomedical Ontology
OWA: Open world assumption
OWL: Web ontology language
OWL-API: Web ontology language application-programming interface
SNOMED: Systematized Nomenclature of Medicine
SWRL: Semantic web rue language
TNM: TNM classification of malignant tumours (T: size of original tumour; N: involvement of regional lymph nodes; M: presence of distant metastasis)
UICC: International Union Against Cancer
